# Abundance of non-native crabs in intertidal habitats of New England with natural and artificial structure

**DOI:** 10.7717/peerj.1246

**Published:** 2015-09-10

**Authors:** Christina M. Lovely, Nancy J. O’Connor, Michael L. Judge

**Affiliations:** 1 Department of Biology, University of Massachusetts Dartmouth, N. Dartmouth, MA, USA; 2 Department of Biology, Manhattan College, Riverdale, NY, USA

**Keywords:** *Carcinus maenas*, Green crab, *Hemigrapsus sanguineus*, Asian shore crab, Density, Habitat structure, Oyster

## Abstract

Marine habitats containing complex physical structure (e.g., crevices) can provide shelter from predation for benthic invertebrates. To examine effects of natural and artificial structure on the abundance of intertidal juvenile crabs, 2 experiments were conducted in Kingston Bay, Massachusetts, USA, from July to September, 2012. In the first experiment, structure was manipulated in a two-factor design that was placed in the high intertidal for 3 one-week periods to test for both substrate type (sand vs. rock) and the presence or absence of artificial structure (mesh grow-out bags used in aquaculture, ∼0.5 m^2^ with 62 mm^2^ mesh openings). The Asian shore crab, *Hemigrapsus sanguineus*, and small individuals of the green crab, *Carcinus maenas*, were observed only in the treatments of rocks and mesh bag plus rocks. Most green crabs were small (<6 mm in carapace width) whereas *H. sanguineus* occurred in a wide range of sizes. In the second experiment, 3 levels of oyster-shell treatments were established using grow-out bags placed on a muddy sand substrate in the low intertidal zone: mesh grow-out bags without shells, grow-out bags with oyster shells, and grow-out bags containing live oysters. Replicate bags were deployed weekly for 7 weeks in a randomized complete block design. All crabs collected in the bags were juvenile *C. maenas* (1–15 mm carapace width), and numbers of crabs differed 6-fold among treatments, with most crabs present in bags with live oysters (29.5 ± 10.6 m^−2^ [mean ± S.D.]) and fewest in bags without shells (4.9 ± 3.7 m^−2^). Both *C. maenas* and *H. sanguineus* occurred in habitats with natural structure (cobble rocks). The attraction of juvenile *C. maenas* to artificial structure consisting of plastic mesh bags containing both oyster shells and living oysters could potentially impact oyster aquaculture operations.

## Introduction

Benthic (bottom-dwelling) marine invertebrates are often associated with complex habitats containing rocks, vegetation, mollusk shells, and other three-dimensional structure. Along with providing potential food resources, structurally complex habitats may confer protection from predation. For example, small juvenile lobsters (*Homarus americanus*) are found primarily in cobble rock habitats in the Gulf of Maine (Wahle & Steneck, 1991), where they are protected from predation by demersal fishes and crabs (Wahle & Steneck, 1992). Limited availability of structured habitats that provide shelter from predators can create demographic bottlenecks in recruitment to crustacean populations (Wahle & Steneck, 1991; Beck, 1995).

Along rocky shores of northeastern North America, two of the most common species of crab are both non-native: the green or shore crab *Carcinus maenas* (Linnaeus, 1758) and the Asian shore crab *Hemigrapsus sanguineus* (de Haan, 1835). The invasion of *C. maenas* occurred in the early 1800s (Behrens Yamada, 2001) whereas *H. sanguineus* became established in the 1980s (Epifanio, 2013). Currently, *C. maenas* occurs from New Jersey to Prince Edward Island (Behrens Yamada, 2001) and the range of *H. sanguineus* extends from North Carolina to Maine (Epifanio, 2013). The biology of *C. maenas* in its native range, Europe, has been well studied, but less information is known about its early life history in North America. In contrast, *H. sanguineus* has been the focus of numerous studies in North America but its biology in the western North Pacific, its native range, is less well known.

In Europe, newly-settled green crabs occur primarily in intertidal and shallow subtidal habitats containing natural or biogenic structure, especially mussel and eelgrass beds and areas with macroalgae (Klein Breteler, 1976; Eriksson & Edlund, 1977; Pihl & Rosenberg, 1982; Thiel & Dernedde, 1994; Hedvall, Moksnes & Pihl, 1998; Moksnes, 2002). In Europe and North America, older juvenile and adult *C. maenas* move to primarily subtidal habitats (Edwards, 1958; Naylor, 1962; Berrill, 1982; Pihl & Rosenberg, 1982). In both its native (western North Pacific) and non-native (North America) ranges, all stages of the Asian shore crab primarily inhabit rocky intertidal shorelines (Lohrer et al., 2000a; Kraemer et al., 2007; O’Connor, 2014), although they can occasionally be found in biogenic structure (Altieri et al., 2010) and appear to move to shallow subtidal habitats to over-winter (Gilman & Grace, 2009).

Both *C. maenas* and *H. sanguineus* are opportunistic omnivores that eat bivalves as well as other invertebrates and macroalgae (Ropes, 1968; Elner, 1981; Lohrer et al., 2000b; Ledesma & O’Connor, 2001; Bourdeau & O’Connor, 2003; Brousseau, Filipowicz & Baglivo, 2005), although *C. maenas* is considered to have a greater negative impact on commercially important bivalves such as clams and oysters (White & Wilson, 1996; Floyd & Williams, 2004; Mascaro & Seed, 2001). Crabs therefore may be attracted to bivalves both for food and protection from predation.

Although *C. maenas* is a relatively long-term resident of northeastern North America, quantitative information about the abundance of juvenile green crabs (<30 mm carapace width) in North American intertidal habitats is limited to work by Berrill (1982), Cameron & Metaxas (2005), and Donahue et al. (2009). With the invasion of *H. sanguineus*, more studies of intertidal crab populations in natural habitats, especially in New England, were initiated, and some have documented the decline of *C. maenas* in rocky intertidal areas where *H. sanguineus* is abundant (Lohrer & Whitlatch, 2002; O’Connor, 2014). Yet relatively few studies have shown co-occurrence after both invaders clearly established residence in the intertidal zone.

Both *C. maenas* and *H. sanguineus* appear to be attracted to habitats with 3-dimensional structure and may compete for shelter (Jensen, McDonald & Armstrong, 2002). The goal of the present study was to compare abundances of the non-native crabs *C. maenas* and *H. sanguineus* in plots containing natural and artificial structure. Natural structure consisted of cobble-sized (6–25 cm) rocks and artificial structure was created using plastic mesh bags commonly employed in oyster aquaculture.

## Materials & Methods

### Location of study site

**Figure 1:**
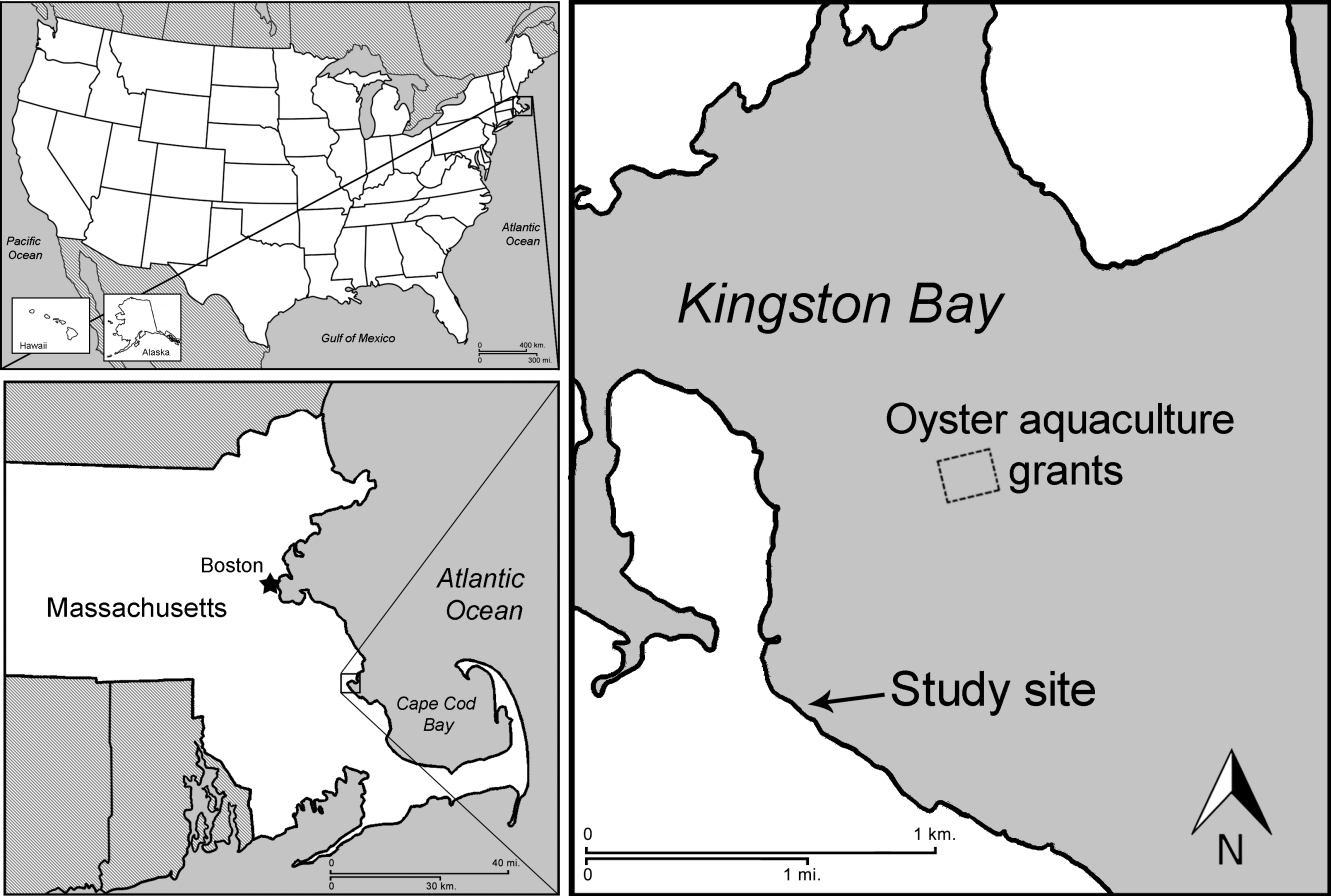
Location of field experiments. Map of study site in Kingston Bay, Massachusetts.

Experiments were conducted in Kingston Bay, Massachusetts (41°59′09.9″N, 70°41′49.7″W) (Fig. 1), a shallow, protected embayment of Cape Cod Bay with both rocky intertidal shoreline and muddy sand flats and a tidal range of approximately 2.4 m. An area permitted for oyster aquaculture was located approximately 1.6 km from the study site. Two experimental sites were chosen within the bay: (1) the high intertidal zone (approx. 1.7 m above mean low water, MLW), with a mix of coarse sand and pebble substrate, as well as patches of cobble rocks; and (2) the low intertidal zone (approx. 26 cm above MLW) with a muddy sand substrate devoid of complex natural structure to separate the effects of natural vs. artificial structure. Experiments were conducted from late June to September, 2012, during which time juvenile crabs were expected to be present (Williams, 1984). Air temperature ranged from 12 to 27 °C and no major storms occurred.

### General experimental procedures

Black plastic (high density polyethylene, HDPE) bags used for oyster grow-out, measuring 96 cm × 51 cm (∼0.5 m^2^ area) with 62 mm^2^ square mesh openings (1.1 cm maximum diagonal length), were borrowed from local oyster farmers. The bags were pouch-like in shape with a sealable opening at one end. Bags were secured with cable ties to 1 m^2^ frames of 21.3 mm PVC pipe, (holes drilled to sink in seawater and anchored to the substrate using plastic stakes), and placed in the intertidal zone for one week. At the end of each experiment, bags were retrieved at low tide during daylight, and all visible crabs in and under the bags were collected. The bags were placed into clean plastic bins and thoroughly rinsed in fresh water to remove crabs. The rinse water was collected and strained through a 500 µm mesh sieve and crabs were frozen until analysis. All crabs collected were identified to species using morphological descriptions in Shen (1935), Gosner (1978), Hogarth (1978), and Williams (1984), and the carapace width (CW, measured at the two widest points of the carapace) was determined using a ruler for large crabs and an ocular micrometer for small crabs. The research was conducted under scientific permit number 054542 issued by the Commonwealth of Massachusetts Division of Marine Fisheries.

### Experiment 1: grow-out bags with natural substrate

A three-factor experimental design tested the effects of natural substrate (sand or rocks), mesh grow-out bags (present or absent) and sampling period on crab abundance. Study plots with all treatments (sand, rocks, sand covered by mesh bag, and rocks covered by mesh bag) were established in the high intertidal zone on 6 July 2012. Each treatment, replicated twice, was deployed for 6 days, and the experiment was repeated for 3 consecutive weeks. At the end of each 6-d deployment, all materials (mesh bags, rocks) were removed to collect crabs. In all ∼0.5 m^2^ plots, approximately 2.5 cm of sand to a depth of approx. 2.5 cm was examined to collect buried crabs.

### Experiment 2: grow-out bags with bivalves

The second experiment tested the effect of grow-out bags containing bivalves on crab abundance on muddy sand substrate. The experimental design comprised 3 levels of bivalve shell status (mesh bag without oyster shells, mesh bag containing aged shells without oyster flesh, and mesh bag with living oysters (cultured *Crassostrea virginica*)) and sampling period. To test for effects of natural, non-living structure on the presence and abundance of crabs, approximately 80 oyster valves in 40 pairs were matched to approximate the surface of a complete oyster. Equal proportions of three size classes of shells (shell length 4–6 cm, 7–8 cm and 9–11 cm) were placed in oyster bags. To test for effects of natural, living structure on the presence and abundance of crabs, approximately 40 oysters in three size classes (shell length 4–6 cm, 7–8 cm and 9–11 cm) in equal proportion were placed in oyster bags. Bags were placed in a line parallel to the water’s edge at low intertidal height. Treatments were arranged randomly in 4 blocks, with each block containing one replicate of each treatment (*n* = 4). Patches of cobble-size rocks occurred 16.2 m towards the north and 9.5 m towards the south.

The experiment was conducted weekly for 7 consecutive weeks, with replicates deployed in a randomized block design over a two-day staggered period beginning 30–31 July 2012. Treatments were left undisturbed for 6 d (as in the previous experiment), then were retrieved on the seventh day and processed as described above. Following processing, bags containing oysters were held in seawater overnight to keep oysters alive before the next deployment. Bags with shell valves remained out of water for 8–9 h between deployments. Retrieved materials were deployed the following day for the next week’s experiment.

### Statistical analysis

In both experiments, crab abundances were analyzed in the R statistical program (R Core Team, 2015) using a generalized linear model assuming a Poisson distribution (with log-link function). GLMER (from the lme4 package) was utilized to test a mixed effect model with experimental treatment as a fixed effect and weekly observation as a random effect. P-values of fixed effects were estimated using drop1 with a likelihood ratio test. Experiment 1 (Grow-out Bags with Natural Substrate) had two fixed factors (substrate (sand/rocks), mesh bag (present/absent)) and weekly observations (3 sampling dates). Because two crab species were collected simultaneously, each crab species was analyzed separately. Experiment 2 (Grow-out Bags with Bivalves) had one fixed factor (bivalve shell status (none/non-living, aged shells/living oysters) plus the random block sampling and weekly observations (7 sampling dates)). Frequency distributions of crab body sizes were analyzed by Kolmogorov–Smirnov two-sample test (K–S) tests to determine if carapace widths differed by crab species (Experiment 1) or bivalve shell status (Experiment 2) (Sokal & Rohlf, 1981). Effect sizes were calculated for treatment comparisons: unstandardized (mean and 95% confidence interval) and standardized (e.g., Cohen’s d with 95% confidence interval, and Pearson r or omega-squared *ω*^2^).

## Results

### Experiment 1: grow-out bags with natural substrate

**Figure 2:**
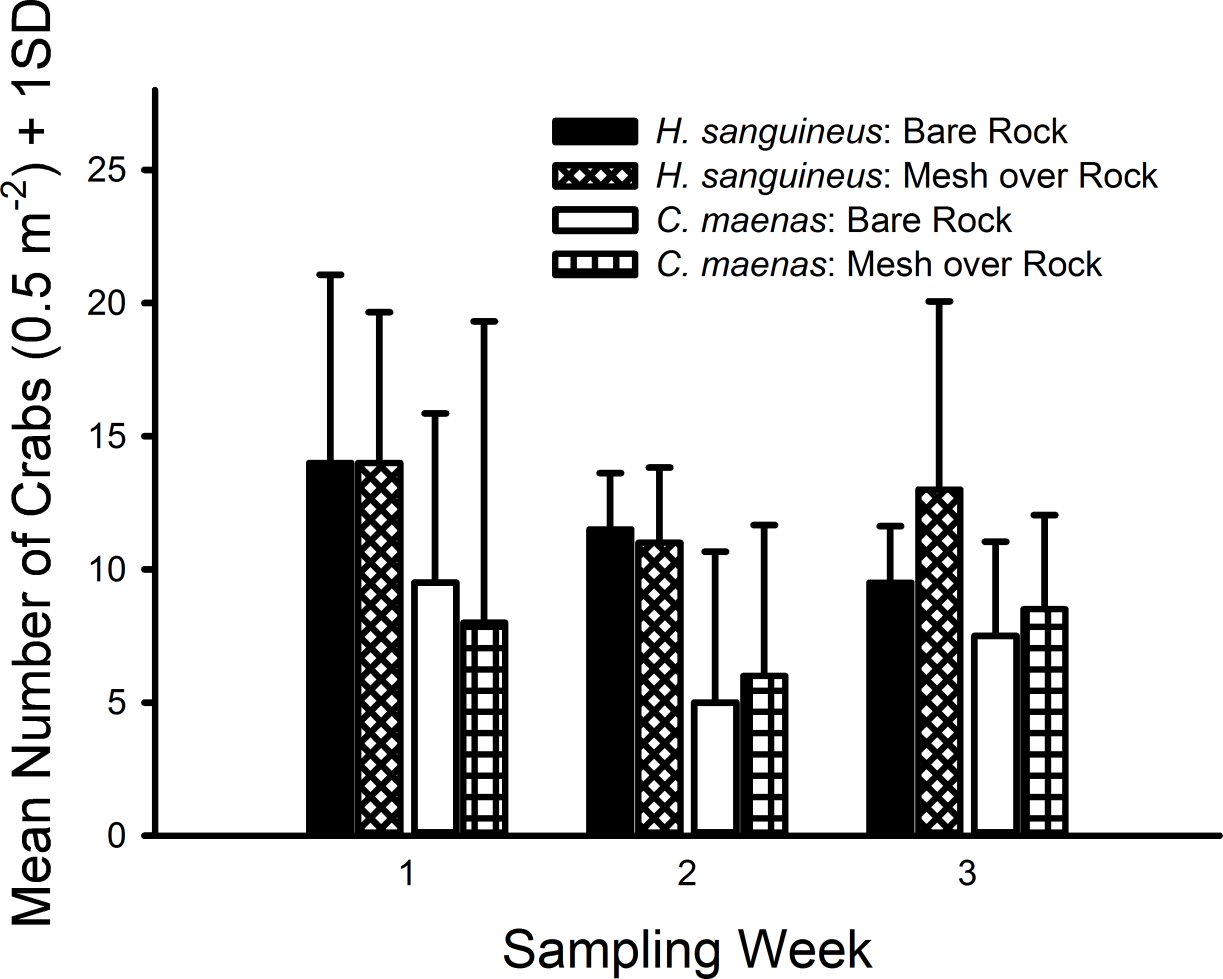
Densities of crabs in rocky substrate 1.7 m above MLW. Mean densities (no. of crabs per 0.5 m^2^) of *Hemigrapsus sanguineus* and *Carcinus maenas* in plots with rocks and rocks with grow-out bags above them. *N* = 2 for each treatment.

In all 3 weekly trials, no crabs were observed in either the bare sand or mesh bag over sand treatments and no crabs were found inside any bags. The green crab, *C. maenas*, and Asian shore crab, *H. sanguineus*, were only observed in the rocks and mesh over rocks treatments (Fig. 2). After removing the sand treatment (both with and without mesh) from the model, crab densities were not measurably affected by the presence of mesh for either *H. sanguineus* (with mesh, mean = 25.33 m^−2^, CI [15.98–34.69] m^−2^; without mesh, mean = 23.33, CI [14.97–31.69]) or *C. maenas* (with mesh, mean = 15.00, CI [1.42–27.58]; without mesh, mean = 14.67, CI [5.03–24.30]). Moreover, both pairwise comparisons possessed small effect sizes: *H. sanguineus* (Cohen’s *d* = 0.237, 95% CI [−0.899–1.372]); *C. maenas* (*d* = 0.031, 95% CI [−1.100–1.163]). Furthermore, each generalized linear model for each species indicated no significant differences in abundance of *H. sanguineus* among mesh treatments (*F*_1,6_ = 0.246, *p* = 0.619), and likewise no significant differences in abundance of *C. maenas* among mesh treatments (*F*_1,6_ = 0.011, *p* = 0.916).

**Figure 3:**
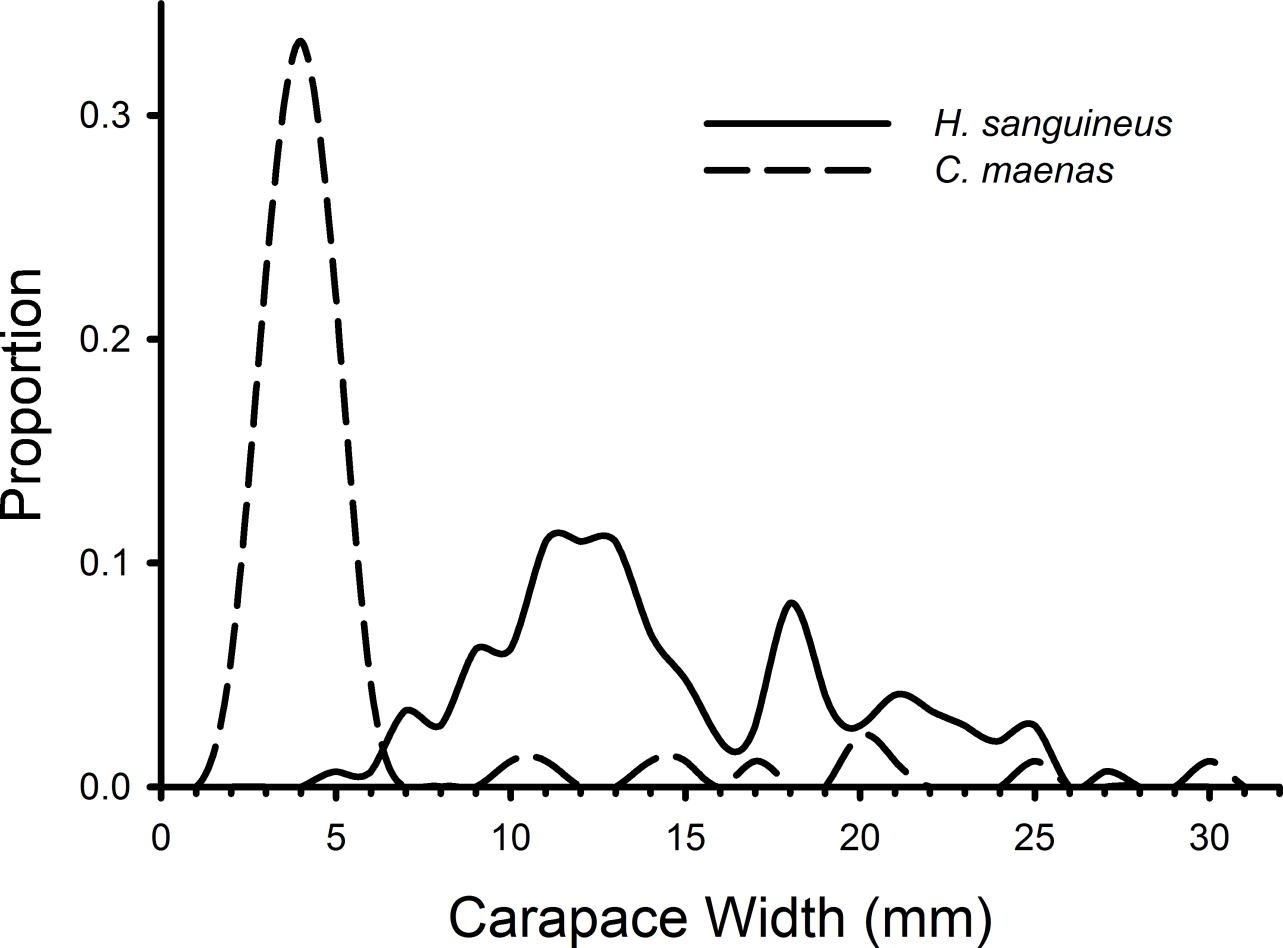
Size frequency distributions of crabs in rocky substrate. The size frequencies of *Hemigrapsus sanguineus* and *Carcinus maenas* in all plots with rocks and rocks with mesh grow-out bags above them pooled for all 3 weeks of the experiment. For *H. sanguineus*, *n* = 146 and for *C. maenas*, *n* = 87.

*H. sanguineus* individuals observed in all treatments during all weeks combined ranged in size from 4 to 27 mm, and *C. maenas* individuals ranged in size from 2 to 30 mm (Fig. 3). The K–S test indicated a significant difference in the size distributions of *C. maenas* and *H. sanguineus* (D = 0.870, *p* < 0.001) and a large effect size (Cohen’s *d* = 1.765, 95% CI [1.455–2.075]). Green crabs (mean = 5.61 mm, 95% CI [4.52–6.70 mm]) were smaller than *H. sanguineus* (mean = 14.62, 95% CI [13.82–15.43]) with 80% of *C. maenas* individuals having a carapace width of 2–5.9 mm (Fig. 3).

### Experiment 2: grow-out bags with bivalves

**Figure 4:**
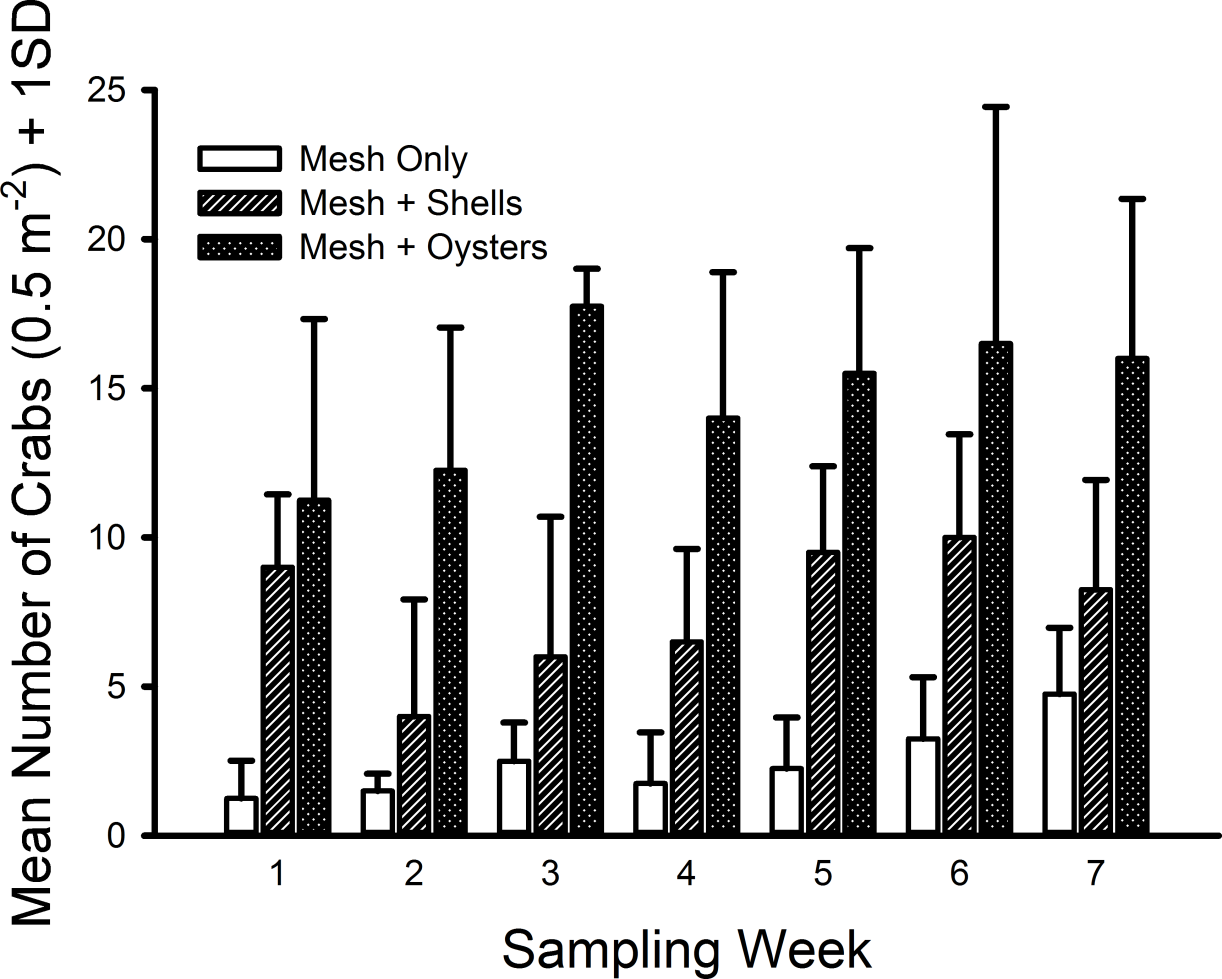
Densities of *Carcinus maenas* in mesh bags 26 cm above MLW. Mean densities (no. of crabs per 0.5 m^2^) of *Carcinus maenas* in each treatment during each week of the experiment testing the effects of mesh grow-out bags with bivalves on crab abundance. *N* = 4 for each treatment in each week.

The only crab species found inside the mesh bags in this experiment was *C. maenas*. The generalized linear model indicated significant differences existed among shell status treatments (*F*_2,59_ = 106.699, *p* ≪ 0.001) with a large effect size (*ω*^2^ = 0.666). Crab densities in the mesh-with-living-oysters treatment (mean = 29.50 m^−2^, 95% CI [25.50–33.50] m^−2^) were nearly double that of the mesh-with-shells treatment (mean = 15.21, 95% CI [12.33–18.10]) and six-fold more than the mesh-only treatment (mean = 4.93, 95% CI [3.51–6.35]) (i.e., living oysters > shells > mesh, Fig. 4). Moreover, all three pairwise comparisons possessed large effect sizes: living oysters vs. non-living shells (Cohen’s *d* = 1.561, 95% CI [0.964–2.161], Pearson *r* = 0.615); living oysters vs. mesh (*d* = 3.099, 95% CI [2.327–3.883], *r* = 0.840); non-living shells vs. mesh (*d* = 1.750, 95% CI [1.135–2.368], *r* = 0.658). The block effect was a significant source of variation (*F*_3,59_ = 2.941, *p* = 0.029).

**Figure 5:**
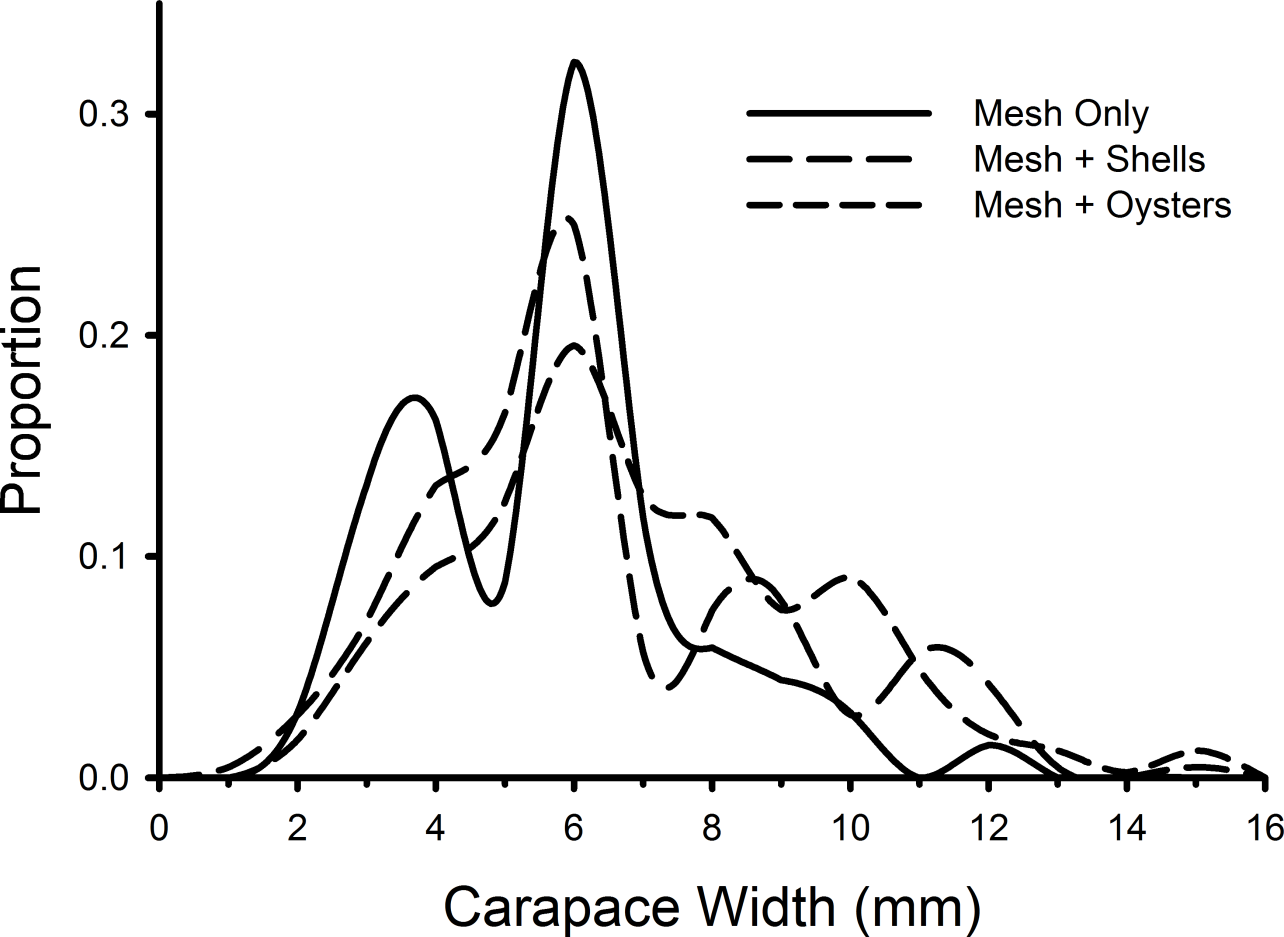
Size frequency distributions of crabs in mesh bags. The size frequencies of *Carcinus maenas* in all plots in each treatment in the experiment testing the effects of mesh grow-out bags with bivalves. For mesh only, *n* = 68; mesh plus shells, *n* = 212; mesh plus oysters *n* = 409.

Individual green crab sizes varied among treatments (Fig. 5). Crabs in the mesh-with-living-oysters (mean = 7.00 mm, 95% CI [6.74–7.25] mm) were significantly larger than those in either the mesh-plus-shells (mean = 6.45, 95% CI [6.09–6.80]; *D* = 0.161, *p* = 0.001) or mesh-alone (mean = 5.66, 95% CI [5.15–6.16]; *D* = 0.264, *p* = 0.001) with small (*d* = 0.218, 95% CI [0.052–0.384]) and medium effect sizes (*d* = 0.538, 95% CI [0.279–0.797]), respectively. There was no significant difference between the sizes of crabs in the mesh-only and mesh-plus-shells treatments (*D* = 0.152, *p* = 0.189). There also was no significant difference in the size distribution of green crabs between the first and last week of the experiment (*D* = 0.117, *p* = 0.512).

## Discussion

Our results confirm the importance of physical structure as habitat for 2 species of non-native crabs, *Hemigrapsus sanguineus* and *Carcinus maenas*, although the species differed in habitat use. Asian shore crabs were found only in rocky habitats; no *H. sanguineus* were found inside any mesh grow-out bags, with or without oysters. Adding additional structure (a grow-out bag) to natural rocky substrate had no effect on *H. sanguineus* density, even though it could have provided a more favorable habitat by shading the area, leading to lower temperatures and less desiccation. Densities of *H. sanguineus* averaged 20–22 crabs/m^2^ (Table 1) and were considerably lower than densities of *H. sanguineus* (80–100 crabs/m^2^) observed at nearby outer coastal locations with extensive rock cover (O’Connor, 2014).

**Table 1:** Densities of crabs in rocky substrate and mesh bags. Average densities (number per m^2^) of Asian shore crabs (*Hemigrapsus sanguineus*) and green crabs (*Carcinus maenas*) in each treatment, all weeks combined, in both experiments.

	Experiment 1				Experiment 2					
Species	Rocks		Mesh + Rocks		Mesh		Mesh + Shells		Mesh + Oysters	
	Mean	SD	Mean	SD	Mean	SD	Mean	SD	Mean	SD
*H. sanguineus*	20.2	9.2	22.2	10.2	0	0	0	0	0	0
*C. maenas*	11.8	9.5	14.3	10.9	5.5	3.4	15.8	7.0	29.5	10.3

Green crabs (*C. maenas*) occurred in upper-intertidal rocky habitats. However, unlike *H. sanguineus*, the overwhelming majority (90%) of *C. maenas* were small (<10 mm CW), which is consistent with prior work showing that the upper as well as the lower rocky intertidal zone is a location of green crab recruitment (Zeng, Abello & Naylor, 1999). Densities of *C. maenas* in the rock treatments (12–14 crabs/m^2^, Table 1) were similar to densities in nearby rocky coastal areas before *H. sanguineus* increased greatly in number (O’Connor, 2014).

Juvenile green crabs rapidly colonized (within 6 days) the mesh bags placed on muddy sand in the low intertidal zone, although few *C. maenas* were found in the empty mesh grow-out bags. The highest numbers of green crabs were consistently observed in bags containing living oysters. This result differs from the findings of Tolley & Volety (2005) who observed similar numbers of decapods (crab and shrimp) in clean shells and live oysters in the field for 30 days in Florida. In their study, both shell and live oyster substrates contained more decapods than bare sand bottom. Nonetheless, oyster beds, owing to their 3-dimensional structure, have been generally shown to be important habitats for decapods (Posey, Alphin & Powell, 1999). While we are unable to compare our results with crab abundance in oyster reefs near our site, Marenghi et al. (2010) found that mesh bags and floating cages attracted invertebrates and fishes similarly to created oyster reefs.

In our experiment, the 80 valves from dead oysters likely provided more structural complexity and refuge from predation than the 40 intact oysters in a bag (Humphries, La Peyre & Decossas, 2011). Because more green crabs were found in bags with living oysters than oyster shells, they were probably responding to chemical cues released from living oysters, or other organisms living on the oysters, rather than to physical refuge from predators alone (Weissburg & Zimmer-Faust, 1994; Weissburg et al., 2002). Although only small <10 mm CW) *C. maenas* were able to enter the bag through the mesh openings, we observed slightly larger green crabs in the bags with live oysters. This result suggests that either larger crabs may be more attracted to living oysters than empty shells, or larger crabs within the bags experience less mortality. Furthermore, once inside the bags with living oysters, crabs might experience faster growth rates. In Sweden, green crabs can reach 9 mm CW or more in their first year (Eriksson & Edlund, 1977), and growth rates might be faster in warmer water, decreasing the time required for crabs to reach a size at which they can open or crush small oysters.

A surprising finding was that no mud crabs (family Panopeidae) were found in any of the mesh bags in the low intertidal zone, even those containing living oysters. Mud crabs are common in intertidal and subtidal estuarine habitats (Ryan, 1956; Meyer, 1994). In addition, small mud crabs are often associated with rocks and bivalves (Lindsey, Altieri & Witman, 2006; Moksnes & Heck, 2006) and are known predators of oysters (Elner & Lavoie, 1983; Kulp et al., 2011). It is possible that habitat patches with mud crabs were too distant from the mesh bags for the mud crabs to easily colonize the bags. The bags might have also been too far from rocky areas for *H. sanguineus* to move to them.

Both green crabs (*C. maenas*) and Asian shore crabs (*H. sanguineus*) occurred in intertidal habitats with physical structure. Although *H. sanguineus* was found only in rocky habitat, *C. maenas* occurred both in rocky areas as well as in artificial structure (mesh grow-out bags used in aquaculture), especially when the bags contained oyster shells or living oysters. Even though bags were in the field for only 6 days, they attracted juvenile green crabs in densities similar to or higher than those in natural rocky habitats, and might serve as additional habitats for recruitment. The attraction of potential crab predators to oyster shells could potentially impact oyster aquaculture if shells are used as a substrate for juvenile oyster cultivation.

## Supplemental Information

10.7717/peerj.1246/supp-1 Supplemental Information 1 Data for experiment 1

10.7717/peerj.1246/supp-2 Supplemental Information 2 Data for experiment 2

## Additional Information and Declarations

### Field Study Permissions

The following information was supplied relating to field study approvals (i.e., approving body and any reference numbers): Commonwealth of Massachusetts Division of Marine Fisheries Scientific permit number 054542.
