# Supplementary material for: Abundance of non-native crabs in intertidal habitats of New England with natural and artificial structure
Source: PeerJ. 2015 Sep 10;3:e1246. doi: 10.7717/peerj.1246 (PMC4579033; doi:10.7717/peerj.1246)
Supplement: Supplemental Information 1 [file peerj-03-1246-s001.pdf]

DATE	TREATMENT	REPLICATE	SPECIES	CARAPACE WIDTH (mm)
6/27/2012	Rock	1	Hemigrapsus	17.0
6/27/2012	Rock	1	Hemigrapsus	11.3
6/27/2012	Rock	1	Hemigrapsus	9.7
6/27/2012	Rock	1	Hemigrapsus	8.2
6/27/2012	Rock	1	Hemigrapsus	9.1
6/27/2012	Rock	1	Hemigrapsus	8.2
6/27/2012	Rock	1	Hemigrapsus	5.8
6/27/2012	Rock	1	Hemigrapsus	8.0
6/27/2012	Rock	1	Carcinus	23.0
6/27/2012	RockMesh	1	Hemigrapsus	24.0
6/27/2012	RockMesh	1	Hemigrapsus	25.0
6/27/2012	RockMesh	1	Hemigrapsus	17.0
6/27/2012	RockMesh	1	Hemigrapsus	10.0
6/27/2012	RockMesh	1	Hemigrapsus	4.7
6/27/2012	RockMesh	1	Hemigrapsus	11.2
6/27/2012	RockMesh	1	Hemigrapsus	10.5
6/27/2012	RockMesh	1	Hemigrapsus	11.0
6/27/2012	RockMesh	1	Hemigrapsus	7.7
6/27/2012	RockMesh	1	Hemigrapsus	9.0
6/27/2012	RockMesh	1	Carcinus	12.0
7/5/2012	Rock	1	Hemigrapsus	14.1
7/5/2012	Rock	1	Hemigrapsus	12.5
7/5/2012	Rock	1	Hemigrapsus	12.2
7/5/2012	Rock	1	Carcinus	24.0
7/5/2012	Rock	1	Carcinus	9.0
7/5/2012	RockMesh	1	Hemigrapsus	13.4
7/5/2012	RockMesh	1	Hemigrapsus	13.4
7/5/2012	RockMesh	1	Hemigrapsus	8.5
7/5/2012	RockMesh	1	Carcinus	33.0
7/5/2012	RockMesh	1	Carcinus	19.0
7/5/2012	RockMesh	1	Carcinus	17.0
7/5/2012	RockMesh	1	Carcinus	11.8
7/12/2012	Rock	1	Hemigrapsus	6.8
7/12/2012	Rock	1	Hemigrapsus	8.4
7/12/2012	Rock	1	Hemigrapsus	8.3
7/12/2012	Rock	1	Hemigrapsus	9.2
7/12/2012	Rock	1	Hemigrapsus	9.6
7/12/2012	Rock	1	Hemigrapsus	6.9
7/12/2012	Rock	1	Hemigrapsus	7.4
7/12/2012	Rock	1	Hemigrapsus	7.7
7/12/2012	Rock	1	Hemigrapsus	10.5
7/12/2012	Rock	1	Hemigrapsus	13.3
7/12/2012	Rock	1	Hemigrapsus	13.1
7/12/2012	Rock	1	Hemigrapsus	14.0
7/12/2012	Rock	1	Hemigrapsus	13.2

7/12/2012	Rock	1	Hemigrapsus	13.5
7/12/2012	Rock	1	Hemigrapsus	15.5
7/12/2012	Rock	1	Hemigrapsus	18.0
7/12/2012	Rock	1	Hemigrapsus	18.5
7/12/2012	Rock	1	Hemigrapsus	18.0
7/12/2012	Rock	1	Hemigrapsus	18.5
7/12/2012	Rock	1	Carcinus	10.3
7/12/2012	Rock	1	Carcinus	17.0
7/12/2012	Rock	1	Carcinus	14.0
7/12/2012	Rock	1	Carcinus	15.5
7/12/2012	Rock	1	Carcinus	20.5
7/12/2012	Rock	1	Carcinus	4.3
7/12/2012	Rock	1	Carcinus	4.0
7/12/2012	Rock	1	Carcinus	3.7
7/12/2012	Rock	1	Carcinus	3.6
7/12/2012	Rock	1	Carcinus	3.5
7/12/2012	Rock	1	Carcinus	2.9
7/12/2012	Rock	1	Carcinus	3.8
7/12/2012	Rock	1	Carcinus	2.8
7/12/2012	Rock	1	Carcinus	2.8
7/12/2012	Rock	2	Hemigrapsus	9.9
7/12/2012	Rock	2	Hemigrapsus	10.8
7/12/2012	Rock	2	Hemigrapsus	11.9
7/12/2012	Rock	2	Hemigrapsus	11.3
7/12/2012	Rock	2	Hemigrapsus	12.3
7/12/2012	Rock	2	Hemigrapsus	13.5
7/12/2012	Rock	2	Hemigrapsus	13.6
7/12/2012	Rock	2	Hemigrapsus	18.0
7/12/2012	Rock	2	Hemigrapsus	21.5
7/12/2012	Rock	2	Carcinus	3.8
7/12/2012	Rock	2	Carcinus	3.9
7/12/2012	Rock	2	Carcinus	11.4
7/12/2012	Rock	2	Carcinus	5.4
7/12/2012	Rock	2	Carcinus	25.2
7/12/2012	RockMesh	1	Hemigrapsus	5.9
7/12/2012	RockMesh	1	Hemigrapsus	8.3
7/12/2012	RockMesh	1	Hemigrapsus	9.4
7/12/2012	RockMesh	1	Hemigrapsus	10.3
7/12/2012	RockMesh	1	Hemigrapsus	9.5
7/12/2012	RockMesh	1	Hemigrapsus	11.2
7/12/2012	RockMesh	1	Hemigrapsus	10.6
7/12/2012	RockMesh	1	Hemigrapsus	12.5
7/12/2012	RockMesh	1	Hemigrapsus	12.5
7/12/2012	RockMesh	1	Hemigrapsus	13.4
7/12/2012	RockMesh	1	Hemigrapsus	12.9
7/12/2012	RockMesh	1	Hemigrapsus	14.2
7/12/2012	RockMesh	1	Hemigrapsus	15.0

7/12/2012	RockMesh	1	Hemigrapsus	16.5
7/12/2012	RockMesh	1	Hemigrapsus	19.4
7/12/2012	RockMesh	1	Hemigrapsus	25.0
7/12/2012	RockMesh	1	Hemigrapsus	24.8
7/12/2012	RockMesh	1	Hemigrapsus	4.9
7/12/2012	RockMesh	1	Carcinus	19.8
7/12/2012	RockMesh	1	Carcinus	2.7
7/12/2012	RockMesh	1	Carcinus	3.8
7/12/2012	RockMesh	1	Carcinus	3.7
7/12/2012	RockMesh	1	Carcinus	3.8
7/12/2012	RockMesh	1	Carcinus	3.0
7/12/2012	RockMesh	1	Carcinus	2.8
7/12/2012	RockMesh	1	Carcinus	4.0
7/12/2012	RockMesh	1	Carcinus	4.0
7/12/2012	RockMesh	1	Carcinus	3.2
7/12/2012	RockMesh	1	Carcinus	3.7
7/12/2012	RockMesh	1	Carcinus	3.7
7/12/2012	RockMesh	1	Carcinus	3.6
7/12/2012	RockMesh	1	Carcinus	3.7
7/12/2012	RockMesh	1	Carcinus	3.6
7/12/2012	RockMesh	1	Carcinus	3.8
7/12/2012	RockMesh	2	Hemigrapsus	9.4
7/12/2012	RockMesh	2	Hemigrapsus	10.0
7/12/2012	RockMesh	2	Hemigrapsus	10.6
7/12/2012	RockMesh	2	Hemigrapsus	12.0
7/12/2012	RockMesh	2	Hemigrapsus	12.3
7/12/2012	RockMesh	2	Hemigrapsus	14.5
7/12/2012	RockMesh	2	Hemigrapsus	14.4
7/12/2012	RockMesh	2	Hemigrapsus	14.2
7/12/2012	RockMesh	2	Hemigrapsus	14.9
7/12/2012	RockMesh	2	Hemigrapsus	19.9
7/19/2012	Rock	1	Hemigrapsus	10.5
7/19/2012	Rock	1	Hemigrapsus	9.1
7/19/2012	Rock	1	Hemigrapsus	11.6
7/19/2012	Rock	1	Hemigrapsus	10.6
7/19/2012	Rock	1	Hemigrapsus	13.5
7/19/2012	Rock	1	Hemigrapsus	13.2
7/19/2012	Rock	1	Hemigrapsus	18.3
7/19/2012	Rock	1	Hemigrapsus	15.8
7/19/2012	Rock	1	Hemigrapsus	17.6
7/19/2012	Rock	1	Hemigrapsus	21.3
7/19/2012	Rock	1	Hemigrapsus	22.0
7/19/2012	Rock	1	Hemigrapsus	23.0
7/19/2012	Rock	1	Hemigrapsus	23.9
7/19/2012	Rock	1	Carcinus	2.8
7/19/2012	Rock	2	Hemigrapsus	9.2
7/19/2012	Rock	2	Hemigrapsus	11.9

7/19/2012	Rock	2	Hemigrapsus	11.6
7/19/2012	Rock	2	Hemigrapsus	12.0
7/19/2012	Rock	2	Hemigrapsus	11.5
7/19/2012	Rock	2	Hemigrapsus	13.2
7/19/2012	Rock	2	Hemigrapsus	13.1
7/19/2012	Rock	2	Hemigrapsus	18.9
7/19/2012	Rock	2	Hemigrapsus	23.3
7/19/2012	Rock	2	Hemigrapsus	21.8
7/19/2012	Rock	2	Carcinus	29.9
7/19/2012	Rock	2	Carcinus	2.7
7/19/2012	Rock	2	Carcinus	4.6
7/19/2012	Rock	2	Carcinus	5.0
7/19/2012	Rock	2	Carcinus	5.0
7/19/2012	Rock	2	Carcinus	4.4
7/19/2012	Rock	2	Carcinus	4.8
7/19/2012	Rock	2	Carcinus	4.3
7/19/2012	Rock	2	Carcinus	3.3
7/19/2012	RockMesh	1	Hemigrapsus	8.7
7/19/2012	RockMesh	1	Hemigrapsus	9.2
7/19/2012	RockMesh	1	Hemigrapsus	12.2
7/19/2012	RockMesh	1	Hemigrapsus	14.1
7/19/2012	RockMesh	1	Hemigrapsus	17.9
7/19/2012	RockMesh	1	Hemigrapsus	18.8
7/19/2012	RockMesh	1	Hemigrapsus	21.5
7/19/2012	RockMesh	1	Hemigrapsus	22.8
7/19/2012	RockMesh	1	Hemigrapsus	27.5
7/19/2012	RockMesh	1	Carcinus	5.1
7/19/2012	RockMesh	1	Carcinus	4.6
7/19/2012	RockMesh	1	Carcinus	3.8
7/19/2012	RockMesh	1	Carcinus	4.0
7/19/2012	RockMesh	1	Carcinus	5.2
7/19/2012	RockMesh	1	Carcinus	4.3
7/19/2012	RockMesh	1	Carcinus	3.5
7/19/2012	RockMesh	1	Carcinus	3.6
7/19/2012	RockMesh	2	Hemigrapsus	11.8
7/19/2012	RockMesh	2	Hemigrapsus	10.9
7/19/2012	RockMesh	2	Hemigrapsus	11.2
7/19/2012	RockMesh	2	Hemigrapsus	12.0
7/19/2012	RockMesh	2	Hemigrapsus	11.0
7/19/2012	RockMesh	2	Hemigrapsus	13.4
7/19/2012	RockMesh	2	Hemigrapsus	14.5
7/19/2012	RockMesh	2	Hemigrapsus	14.4
7/19/2012	RockMesh	2	Hemigrapsus	17.7
7/19/2012	RockMesh	2	Hemigrapsus	19.8
7/19/2012	RockMesh	2	Hemigrapsus	21.7
7/19/2012	RockMesh	2	Hemigrapsus	24.2
7/19/2012	RockMesh	2	Hemigrapsus	25.4

7/19/2012	RockMesh	2	Carcinus	3.5
7/19/2012	RockMesh	2	Carcinus	4.6
7/26/2012	Rock	1	Hemigrapsus	8.9
7/26/2012	Rock	1	Hemigrapsus	10.5
7/26/2012	Rock	1	Hemigrapsus	11.0
7/26/2012	Rock	1	Hemigrapsus	10.6
7/26/2012	Rock	1	Hemigrapsus	14.5
7/26/2012	Rock	1	Hemigrapsus	15.5
7/26/2012	Rock	1	Hemigrapsus	17.4
7/26/2012	Rock	1	Hemigrapsus	16.6
7/26/2012	Rock	1	Hemigrapsus	19.9
7/26/2012	Rock	1	Hemigrapsus	17.9
7/26/2012	Rock	1	Hemigrapsus	16.8
7/26/2012	Rock	1	Carcinus	2.5
7/26/2012	Rock	1	Carcinus	2.6
7/26/2012	Rock	1	Carcinus	3.3
7/26/2012	Rock	1	Carcinus	2.5
7/26/2012	Rock	1	Carcinus	3.9
7/26/2012	Rock	1	Carcinus	4.3
7/26/2012	Rock	1	Carcinus	4.6
7/26/2012	Rock	1	Carcinus	4.5
7/26/2012	Rock	1	Carcinus	5.7
7/26/2012	Rock	1	Carcinus	6.1
7/26/2012	Rock	2	Hemigrapsus	7.2
7/26/2012	Rock	2	Hemigrapsus	9.8
7/26/2012	Rock	2	Hemigrapsus	13.1
7/26/2012	Rock	2	Hemigrapsus	11.6
7/26/2012	Rock	2	Hemigrapsus	12.2
7/26/2012	Rock	2	Hemigrapsus	12.6
7/26/2012	Rock	2	Hemigrapsus	17.6
7/26/2012	Rock	2	Hemigrapsus	22.0
7/26/2012	Rock	2	Carcinus	2.5
7/26/2012	Rock	2	Carcinus	3.5
7/26/2012	Rock	2	Carcinus	3.3
7/26/2012	Rock	2	Carcinus	5.6
7/26/2012	Rock	2	Carcinus	4.6
7/26/2012	RockMesh	1	Hemigrapsus	7.2
7/26/2012	RockMesh	1	Hemigrapsus	10.9
7/26/2012	RockMesh	1	Hemigrapsus	11.5
7/26/2012	RockMesh	1	Hemigrapsus	11.3
7/26/2012	RockMesh	1	Hemigrapsus	10.5
7/26/2012	RockMesh	1	Hemigrapsus	12.8
7/26/2012	RockMesh	1	Hemigrapsus	12.5
7/26/2012	RockMesh	1	Hemigrapsus	13.4
7/26/2012	RockMesh	1	Hemigrapsus	14.4
7/26/2012	RockMesh	1	Hemigrapsus	16.0
7/26/2012	RockMesh	1	Hemigrapsus	14.8

7/26/2012	RockMesh	1	Hemigrapsus	18.2
7/26/2012	RockMesh	1	Hemigrapsus	18.8
7/26/2012	RockMesh	1	Hemigrapsus	20.3
7/26/2012	RockMesh	1	Hemigrapsus	21.2
7/26/2012	RockMesh	1	Hemigrapsus	22.9
7/26/2012	RockMesh	1	Hemigrapsus	22.0
7/26/2012	RockMesh	1	Hemigrapsus	24.7
7/26/2012	RockMesh	1	Carcinus	2.5
7/26/2012	RockMesh	1	Carcinus	2.5
7/26/2012	RockMesh	1	Carcinus	2.6
7/26/2012	RockMesh	1	Carcinus	3.1
7/26/2012	RockMesh	1	Carcinus	3.4
7/26/2012	RockMesh	1	Carcinus	4.6
7/26/2012	RockMesh	1	Carcinus	4.6
7/26/2012	RockMesh	1	Carcinus	5.1
7/26/2012	RockMesh	1	Carcinus	4.3
7/26/2012	RockMesh	1	Carcinus	4.7
7/26/2012	RockMesh	1	Carcinus	4.9
7/26/2012	RockMesh	2	Hemigrapsus	11.3
7/26/2012	RockMesh	2	Hemigrapsus	13.9
7/26/2012	RockMesh	2	Hemigrapsus	16.7
7/26/2012	RockMesh	2	Hemigrapsus	18.9
7/26/2012	RockMesh	2	Hemigrapsus	19.1
7/26/2012	RockMesh	2	Hemigrapsus	21.4
7/26/2012	RockMesh	2	Hemigrapsus	21.2
7/26/2012	RockMesh	2	Hemigrapsus	23.9
7/26/2012	RockMesh	2	Carcinus	20.8
7/26/2012	RockMesh	2	Carcinus	3.8
7/26/2012	RockMesh	2	Carcinus	4.9
7/26/2012	RockMesh	2	Carcinus	5.8
7/26/2012	RockMesh	2	Carcinus	5.1
7/26/2012	RockMesh	2	Carcinus	5.3
