# Supplementary material for: Abundance of non-native crabs in intertidal habitats of New England with natural and artificial structure
Source: PeerJ. 2015 Sep 10;3:e1246. doi: 10.7717/peerj.1246 (PMC4579033; doi:10.7717/peerj.1246)
Supplement: Supplemental Information 2 [file peerj-03-1246-s002.pdf]

DATE	TREATMENT	REPLICATE	*Carcinus* CARAPACE WIDTH (mm)
8/6/2012	Mesh	1	5.9
8/6/2012	Mesh	1	5.0
8/6/2012	Mesh	1	6.3
8/7/2012	Mesh	3	5.6
8/7/2012	Mesh	4	3.4
8/6/2012	Shells	1	11.2
8/6/2012	Shells	1	4.9
8/6/2012	Shells	1	6.7
8/6/2012	Shells	1	11.9
8/6/2012	Shells	1	6.1
8/6/2012	Shells	1	2.9
8/6/2012	Shells	1	6.3
8/6/2012	Shells	1	5.4
8/6/2012	Shells	1	3.6
8/6/2012	Shells	2	3.8
8/6/2012	Shells	2	3.0
8/6/2012	Shells	2	2.6
8/6/2012	Shells	2	6.3
8/6/2012	Shells	2	5.0
8/6/2012	Shells	2	6.2
8/6/2012	Shells	2	2.8
8/6/2012	Shells	2	4.7
8/6/2012	Shells	2	8.5
8/6/2012	Shells	2	4.7
8/6/2012	Shells	2	11.2
8/6/2012	Shells	2	11.6
8/7/2012	Shells	3	9.0
8/7/2012	Shells	3	5.8
8/7/2012	Shells	3	12.3
8/7/2012	Shells	3	6.4
8/7/2012	Shells	3	5.1
8/7/2012	Shells	3	5.7
8/7/2012	Shells	3	3.4
8/7/2012	Shells	3	9.1
8/7/2012	Shells	3	8.3
8/7/2012	Shells	4	6.2
8/7/2012	Shells	4	6.0
8/7/2012	Shells	4	5.8
8/7/2012	Shells	4	4.9
8/7/2012	Shells	4	5.2
8/7/2012	Shells	4	5.7
8/6/2012	Oysters	1	10.9
8/6/2012	Oysters	1	10.9
8/6/2012	Oysters	1	7.6
8/6/2012	Oysters	1	7.5

8/6/2012	Oysters	1	6.2
8/6/2012	Oysters	1	4.6
8/6/2012	Oysters	2	15.0
8/6/2012	Oysters	2	15.0
8/6/2012	Oysters	2	8.4
8/6/2012	Oysters	2	6.3
8/6/2012	Oysters	2	6.7
8/6/2012	Oysters	2	6.5
8/7/2012	Oysters	3	12.8
8/7/2012	Oysters	3	6.4
8/7/2012	Oysters	3	5.7
8/7/2012	Oysters	3	10.6
8/7/2012	Oysters	3	6.1
8/7/2012	Oysters	3	6.0
8/7/2012	Oysters	3	4.6
8/7/2012	Oysters	3	6.6
8/7/2012	Oysters	3	4.5
8/7/2012	Oysters	3	6.1
8/7/2012	Oysters	3	5.2
8/7/2012	Oysters	3	5.5
8/7/2012	Oysters	3	4.8
8/7/2012	Oysters	3	6.2
8/7/2012	Oysters	3	5.4
8/7/2012	Oysters	3	5.9
8/7/2012	Oysters	4	2.9
8/7/2012	Oysters	4	15.0
8/7/2012	Oysters	4	15.0
8/7/2012	Oysters	4	5.6
8/7/2012	Oysters	4	4.4
8/7/2012	Oysters	4	4.8
8/7/2012	Oysters	4	6.2
8/7/2012	Oysters	4	2.8
8/7/2012	Oysters	4	5.9
8/7/2012	Oysters	4	5.1
8/7/2012	Oysters	4	5.6
8/7/2012	Oysters	4	6.5
8/7/2012	Oysters	4	4.6
8/7/2012	Oysters	4	6.2
8/7/2012	Oysters	4	7.0
8/7/2012	Oysters	4	6.2
8/14/2012	Mesh	1	1.9
8/14/2012	Mesh	1	2.8
8/14/2012	Mesh	2	5.8
8/15/2012	Mesh	3	5.7
8/15/2012	Mesh	4	6.2
8/14/2012	Shells	1	2.8
8/14/2012	Shells	1	4.1

8/14/2012	Shells	1	4.3
8/14/2012	Shells	1	3.4
8/14/2012	Shells	1	6.1
8/14/2012	Shells	1	5.7
8/14/2012	Shells	1	5.4
8/14/2012	Shells	1	5.5
8/14/2012	Shells	1	8.0
8/15/2012	Shells	3	2.1
8/15/2012	Shells	3	3.2
8/15/2012	Shells	3	1.8
8/15/2012	Shells	3	4.3
8/15/2012	Shells	4	2.0
8/15/2012	Shells	4	6.3
8/14/2012	Oysters	1	2.8
8/14/2012	Oysters	1	6.9
8/14/2012	Oysters	1	6.1
8/14/2012	Oysters	1	7.5
8/14/2012	Oysters	1	5.6
8/14/2012	Oysters	1	8.3
8/14/2012	Oysters	1	7.8
8/14/2012	Oysters	2	12.8
8/14/2012	Oysters	2	6.2
8/14/2012	Oysters	2	7.8
8/14/2012	Oysters	2	4.8
8/14/2012	Oysters	2	7.6
8/14/2012	Oysters	2	7.3
8/14/2012	Oysters	2	7.9
8/14/2012	Oysters	2	8.2
8/14/2012	Oysters	2	6.5
8/14/2012	Oysters	2	10.5
8/15/2012	Oysters	3	2.7
8/15/2012	Oysters	3	2.0
8/15/2012	Oysters	3	2.2
8/15/2012	Oysters	3	3.6
8/15/2012	Oysters	3	14.2
8/15/2012	Oysters	3	2.7
8/15/2012	Oysters	3	1.8
8/15/2012	Oysters	3	5.3
8/15/2012	Oysters	3	3.7
8/15/2012	Oysters	3	5.8
8/15/2012	Oysters	3	6.1
8/15/2012	Oysters	3	10.0
8/15/2012	Oysters	3	8.8
8/15/2012	Oysters	3	8.0
8/15/2012	Oysters	3	5.7
8/15/2012	Oysters	3	6.2
8/15/2012	Oysters	3	7.4

8/15/2012	Oysters	3	6.2
8/15/2012	Oysters	4	2.9
8/15/2012	Oysters	4	2.9
8/15/2012	Oysters	4	2.6
8/15/2012	Oysters	4	2.5
8/15/2012	Oysters	4	3.0
8/15/2012	Oysters	4	5.6
8/15/2012	Oysters	4	5.4
8/15/2012	Oysters	4	8.5
8/15/2012	Oysters	4	8.3
8/15/2012	Oysters	4	3.5
8/15/2012	Oysters	4	5.7
8/15/2012	Oysters	4	7.2
8/22/2012	Mesh	1	6.0
8/22/2012	Mesh	1	5.6
8/22/2012	Mesh	2	7.1
8/22/2012	Mesh	2	5.8
8/22/2012	Mesh	2	7.3
8/23/2012	Mesh	3	1.6
8/23/2012	Mesh	3	2.7
8/23/2012	Mesh	3	2.7
8/23/2012	Mesh	3	8.5
8/23/2012	Mesh	4	7.1
8/22/2012	Shells	1	4.3
8/22/2012	Shells	1	7.8
8/22/2012	Shells	1	7.1
8/22/2012	Shells	1	8.3
8/22/2012	Shells	2	3.1
8/22/2012	Shells	2	2.8
8/22/2012	Shells	2	6.0
8/22/2012	Shells	2	6.5
8/22/2012	Shells	2	4.7
8/22/2012	Shells	2	7.9
8/22/2012	Shells	2	6.2
8/22/2012	Shells	2	3.7
8/22/2012	Shells	2	3.7
8/22/2012	Shells	2	4.8
8/22/2012	Shells	2	4.6
8/22/2012	Shells	2	5.5
8/22/2012	Shells	2	10.9
8/23/2012	Shells	3	4.0
8/23/2012	Shells	3	8.0
8/23/2012	Shells	3	7.5
8/23/2012	Shells	4	4.4
8/23/2012	Shells	4	6.0
8/23/2012	Shells	4	5.0
8/23/2012	Shells	4	6.3

8/22/2012	Oysters	1	4.0
8/22/2012	Oysters	1	5.7
8/22/2012	Oysters	1	5.1
8/22/2012	Oysters	1	6.1
8/22/2012	Oysters	1	5.8
8/22/2012	Oysters	1	7.7
8/22/2012	Oysters	1	7.9
8/22/2012	Oysters	1	5.3
8/22/2012	Oysters	1	5.5
8/22/2012	Oysters	1	4.1
8/22/2012	Oysters	1	3.9
8/22/2012	Oysters	1	8.4
8/22/2012	Oysters	1	7.6
8/22/2012	Oysters	1	7.6
8/22/2012	Oysters	1	6.0
8/22/2012	Oysters	1	7.2
8/22/2012	Oysters	2	10.7
8/22/2012	Oysters	2	2.7
8/22/2012	Oysters	2	5.3
8/22/2012	Oysters	2	10.0
8/22/2012	Oysters	2	6.9
8/22/2012	Oysters	2	8.8
8/22/2012	Oysters	2	7.2
8/22/2012	Oysters	2	6.0
8/22/2012	Oysters	2	6.1
8/22/2012	Oysters	2	3.3
8/22/2012	Oysters	2	8.1
8/22/2012	Oysters	2	11.5
8/22/2012	Oysters	2	7.5
8/22/2012	Oysters	2	4.3
8/22/2012	Oysters	2	4.5
8/22/2012	Oysters	2	7.9
8/22/2012	Oysters	2	8.5
8/22/2012	Oysters	2	15.0
8/23/2012	Oysters	3	3.8
8/23/2012	Oysters	3	2.7
8/23/2012	Oysters	3	3.7
8/23/2012	Oysters	3	2.8
8/23/2012	Oysters	3	5.5
8/23/2012	Oysters	3	7.4
8/23/2012	Oysters	3	5.6
8/23/2012	Oysters	3	6.8
8/23/2012	Oysters	3	8.0
8/23/2012	Oysters	3	8.9
8/23/2012	Oysters	3	7.7
8/23/2012	Oysters	3	8.2
8/23/2012	Oysters	3	8.1

8/23/2012	Oysters	3	9.5
8/23/2012	Oysters	3	9.7
8/23/2012	Oysters	3	11.2
8/23/2012	Oysters	3	9.0
8/23/2012	Oysters	3	8.8
8/23/2012	Oysters	3	10.6
8/23/2012	Oysters	4	4.4
8/23/2012	Oysters	4	2.7
8/23/2012	Oysters	4	3.7
8/23/2012	Oysters	4	2.6
8/23/2012	Oysters	4	3.9
8/23/2012	Oysters	4	5.7
8/23/2012	Oysters	4	6.1
8/23/2012	Oysters	4	7.5
8/23/2012	Oysters	4	5.6
8/23/2012	Oysters	4	7.1
8/23/2012	Oysters	4	8.3
8/23/2012	Oysters	4	5.5
8/23/2012	Oysters	4	6.6
8/23/2012	Oysters	4	6.1
8/23/2012	Oysters	4	7.5
8/23/2012	Oysters	4	6.0
8/23/2012	Oysters	4	9.8
8/23/2012	Oysters	4	7.0
8/30/2012	Mesh	1	3.7
8/30/2012	Mesh	1	7.6
8/30/2012	Mesh	2	9.6
8/31/2012	Mesh	4	3.7
8/31/2012	Mesh	4	3.9
8/31/2012	Mesh	4	5.9
8/31/2012	Mesh	4	9.4
8/30/2012	Shells	1	3.7
8/30/2012	Shells	1	3.8
8/30/2012	Shells	1	3.5
8/30/2012	Shells	1	7.3
8/30/2012	Shells	1	6.5
8/30/2012	Shells	1	7.6
8/30/2012	Shells	1	3.5
8/30/2012	Shells	1	7.5
8/30/2012	Shells	1	11.8
8/30/2012	Shells	1	11.5
8/30/2012	Shells	2	3.3
8/30/2012	Shells	2	3.7
8/30/2012	Shells	2	4.6
8/30/2012	Shells	2	4.8
8/30/2012	Shells	2	4.2
8/30/2012	Shells	2	5.8

8/30/2012	Shells	2	6.2
8/30/2012	Shells	2	10.0
8/31/2012	Shells	3	4.3
8/31/2012	Shells	3	6.0
8/31/2012	Shells	3	8.0
8/31/2012	Shells	3	10.6
8/31/2012	Shells	3	10.0
8/31/2012	Shells	4	2.0
8/31/2012	Shells	4	4.2
8/31/2012	Shells	4	9.2
8/30/2012	Oysters	1	3.5
8/30/2012	Oysters	1	4.4
8/30/2012	Oysters	1	5.8
8/30/2012	Oysters	1	7.2
8/30/2012	Oysters	1	10.5
8/30/2012	Oysters	1	10.0
8/30/2012	Oysters	1	9.4
8/30/2012	Oysters	1	9.2
8/30/2012	Oysters	2	3.2
8/30/2012	Oysters	2	3.6
8/30/2012	Oysters	2	4.7
8/30/2012	Oysters	2	5.2
8/30/2012	Oysters	2	3.5
8/30/2012	Oysters	2	6.9
8/30/2012	Oysters	2	7.4
8/30/2012	Oysters	2	7.5
8/30/2012	Oysters	2	8.6
8/30/2012	Oysters	2	9.9
8/30/2012	Oysters	2	10.0
8/30/2012	Oysters	2	6.8
8/30/2012	Oysters	2	7.9
8/30/2012	Oysters	2	7.4
8/30/2012	Oysters	2	3.6
8/30/2012	Oysters	2	8.1
8/30/2012	Oysters	2	9.3
8/30/2012	Oysters	2	11.3
8/30/2012	Oysters	2	10.6
8/30/2012	Oysters	2	11.9
8/31/2012	Oysters	3	3.7
8/31/2012	Oysters	3	3.6
8/31/2012	Oysters	3	6.7
8/31/2012	Oysters	3	4.8
8/31/2012	Oysters	3	3.9
8/31/2012	Oysters	3	3.5
8/31/2012	Oysters	3	3.8
8/31/2012	Oysters	3	3.8
8/31/2012	Oysters	3	5.6

8/31/2012	Oysters	3	7.6
8/31/2012	Oysters	3	7.5
8/31/2012	Oysters	3	9.3
8/31/2012	Oysters	3	10.0
8/31/2012	Oysters	3	9.8
8/31/2012	Oysters	4	3.8
8/31/2012	Oysters	4	7.4
8/31/2012	Oysters	4	5.9
8/31/2012	Oysters	4	7.1
8/31/2012	Oysters	4	7.2
8/31/2012	Oysters	4	6.0
8/31/2012	Oysters	4	7.9
8/31/2012	Oysters	4	6.3
8/31/2012	Oysters	4	5.6
8/31/2012	Oysters	4	7.9
8/31/2012	Oysters	4	10.0
8/31/2012	Oysters	4	10.9
8/31/2012	Oysters	4	4.5
9/7/2012	Mesh	1	3.4
9/7/2012	Mesh	1	3.7
9/7/2012	Mesh	2	2.7
9/7/2012	Mesh	2	4.1
9/7/2012	Mesh	2	6.9
9/7/2012	Mesh	2	9.3
9/8/2012	Mesh	3	4.3
9/8/2012	Mesh	3	4.6
9/8/2012	Mesh	3	5.9
9/7/2012	Shells	1	4.3
9/7/2012	Shells	1	6.2
9/7/2012	Shells	1	6.3
9/7/2012	Shells	1	5.2
9/7/2012	Shells	1	6.7
9/7/2012	Shells	1	10.1
9/7/2012	Shells	1	6.9
9/7/2012	Shells	1	9.2
9/7/2012	Shells	1	10.5
9/7/2012	Shells	1	12.5
9/7/2012	Shells	2	3.2
9/7/2012	Shells	2	4.1
9/7/2012	Shells	2	4.7
9/7/2012	Shells	2	4.3
9/7/2012	Shells	2	4.1
9/7/2012	Shells	2	9.4
9/8/2012	Shells	3	4.1
9/8/2012	Shells	3	4.5
9/8/2012	Shells	3	6.0
9/8/2012	Shells	3	7.9

9/8/2012	Shells	3	8.2
9/8/2012	Shells	3	9.0
9/8/2012	Shells	3	8.6
9/8/2012	Shells	3	11.9
9/8/2012	Shells	3	1.6
9/8/2012	Shells	4	4.7
9/8/2012	Shells	4	4.7
9/8/2012	Shells	4	5.9
9/8/2012	Shells	4	4.8
9/8/2012	Shells	4	5.8
9/8/2012	Shells	4	7.7
9/8/2012	Shells	4	9.2
9/8/2012	Shells	4	9.4
9/8/2012	Shells	4	8.7
9/8/2012	Shells	4	11.5
9/8/2012	Shells	4	11.3
9/8/2012	Shells	4	10.7
9/8/2012	Shells	4	13.1
9/7/2012	Oysters	1	4.2
9/7/2012	Oysters	1	4.0
9/7/2012	Oysters	1	4.3
9/7/2012	Oysters	1	5.0
9/7/2012	Oysters	1	5.7
9/7/2012	Oysters	1	7.1
9/7/2012	Oysters	1	7.0
9/7/2012	Oysters	1	8.1
9/7/2012	Oysters	1	10.5
9/7/2012	Oysters	1	8.0
9/7/2012	Oysters	1	8.6
9/7/2012	Oysters	1	10.5
9/7/2012	Oysters	1	11.3
9/7/2012	Oysters	1	6.8
9/7/2012	Oysters	1	8.3
9/7/2012	Oysters	1	9.4
9/7/2012	Oysters	2	4.6
9/7/2012	Oysters	2	3.5
9/7/2012	Oysters	2	4.3
9/7/2012	Oysters	2	4.6
9/7/2012	Oysters	2	5.9
9/7/2012	Oysters	2	6.0
9/7/2012	Oysters	2	7.6
9/7/2012	Oysters	2	4.3
9/7/2012	Oysters	2	6.2
9/7/2012	Oysters	2	8.0
9/7/2012	Oysters	2	8.9
9/7/2012	Oysters	2	9.8
9/7/2012	Oysters	2	9.7

9/7/2012	Oysters	2	10.1
9/8/2012	Oysters	3	2.5
9/8/2012	Oysters	3	3.9
9/8/2012	Oysters	3	4.6
9/8/2012	Oysters	3	4.7
9/8/2012	Oysters	3	4.8
9/8/2012	Oysters	3	5.2
9/8/2012	Oysters	3	6.7
9/8/2012	Oysters	3	7.1
9/8/2012	Oysters	3	7.0
9/8/2012	Oysters	3	7.6
9/8/2012	Oysters	3	9.7
9/8/2012	Oysters	3	7.8
9/8/2012	Oysters	3	9.3
9/8/2012	Oysters	3	7.5
9/8/2012	Oysters	3	8.1
9/8/2012	Oysters	3	7.9
9/8/2012	Oysters	3	9.6
9/8/2012	Oysters	3	13.2
9/8/2012	Oysters	3	10.9
9/8/2012	Oysters	3	9.3
9/8/2012	Oysters	3	10.1
9/8/2012	Oysters	4	4.5
9/8/2012	Oysters	4	6.9
9/8/2012	Oysters	4	6.9
9/8/2012	Oysters	4	4.4
9/8/2012	Oysters	4	7.2
9/8/2012	Oysters	4	8.7
9/8/2012	Oysters	4	9.2
9/8/2012	Oysters	4	11.3
9/8/2012	Oysters	4	10.9
9/8/2012	Oysters	4	10.0
9/8/2012	Oysters	4	11.3
9/15/2012	Mesh	1	4.5
9/15/2012	Mesh	1	5.3
9/15/2012	Mesh	1	9.8
9/15/2012	Mesh	2	4.5
9/15/2012	Mesh	2	4.4
9/15/2012	Mesh	2	5.8
9/15/2012	Mesh	2	6.2
9/15/2012	Mesh	2	7.5
9/15/2012	Mesh	2	6.6
9/16/2012	Mesh	3	2.7
9/16/2012	Mesh	4	3.6
9/16/2012	Mesh	4	5.9
9/16/2012	Mesh	4	6.3
9/15/2012	Shells	1	7.2

9/15/2012	Shells	1	9.6
9/15/2012	Shells	1	9.4
9/15/2012	Shells	1	5.0
9/15/2012	Shells	1	9.0
9/15/2012	Shells	1	5.4
9/15/2012	Shells	1	9.2
9/15/2012	Shells	1	5.7
9/15/2012	Shells	1	8.0
9/15/2012	Shells	2	6.3
9/15/2012	Shells	2	5.3
9/15/2012	Shells	2	5.4
9/15/2012	Shells	2	5.7
9/15/2012	Shells	2	6.4
9/15/2012	Shells	2	3.9
9/15/2012	Shells	2	4.7
9/15/2012	Shells	2	6.2
9/15/2012	Shells	2	5.7
9/15/2012	Shells	2	7.2
9/15/2012	Shells	2	9.1
9/15/2012	Shells	2	10.6
9/15/2012	Shells	2	11.2
9/15/2012	Shells	2	12.2
9/15/2012	Shells	2	10.9
9/16/2012	Shells	3	4.3
9/16/2012	Shells	3	4.4
9/16/2012	Shells	3	4.7
9/16/2012	Shells	3	5.4
9/16/2012	Shells	3	6.4
9/16/2012	Shells	3	7.2
9/16/2012	Shells	3	9.4
9/16/2012	Shells	4	5.3
9/16/2012	Shells	4	6.1
9/16/2012	Shells	4	5.2
9/16/2012	Shells	4	6.4
9/16/2012	Shells	4	5.7
9/16/2012	Shells	4	7.7
9/16/2012	Shells	4	7.7
9/16/2012	Shells	4	11.3
9/16/2012	Shells	4	6.3
9/15/2012	Oysters	1	5.9
9/15/2012	Oysters	1	5.8
9/15/2012	Oysters	1	7.0
9/15/2012	Oysters	1	8.7
9/15/2012	Oysters	1	9.2
9/15/2012	Oysters	1	10.9
9/15/2012	Oysters	2	4.0
9/15/2012	Oysters	2	4.7

9/15/2012	Oysters	2	5.3
9/15/2012	Oysters	2	6.4
9/15/2012	Oysters	2	7.4
9/15/2012	Oysters	2	7.4
9/15/2012	Oysters	2	7.3
9/15/2012	Oysters	2	7.5
9/15/2012	Oysters	2	8.1
9/15/2012	Oysters	2	9.6
9/15/2012	Oysters	2	9.1
9/15/2012	Oysters	2	9.4
9/15/2012	Oysters	2	9.9
9/15/2012	Oysters	2	10.6
9/15/2012	Oysters	2	12.0
9/15/2012	Oysters	2	12.9
9/16/2012	Oysters	3	3.5
9/16/2012	Oysters	3	3.4
9/16/2012	Oysters	3	4.5
9/16/2012	Oysters	3	5.4
9/16/2012	Oysters	3	6.3
9/16/2012	Oysters	3	5.1
9/16/2012	Oysters	3	5.2
9/16/2012	Oysters	3	5.7
9/16/2012	Oysters	3	4.6
9/16/2012	Oysters	3	6.2
9/16/2012	Oysters	3	6.0
9/16/2012	Oysters	3	5.8
9/16/2012	Oysters	3	6.0
9/16/2012	Oysters	3	9.3
9/16/2012	Oysters	3	7.6
9/16/2012	Oysters	3	8.1
9/16/2012	Oysters	3	8.1
9/16/2012	Oysters	3	9.3
9/16/2012	Oysters	3	9.1
9/16/2012	Oysters	3	9.4
9/16/2012	Oysters	3	9.7
9/16/2012	Oysters	3	9.8
9/16/2012	Oysters	3	12.0
9/16/2012	Oysters	3	10.9
9/16/2012	Oysters	3	8.9
9/16/2012	Oysters	4	2.7
9/16/2012	Oysters	4	4.7
9/16/2012	Oysters	4	4.2
9/16/2012	Oysters	4	5.2
9/16/2012	Oysters	4	5.5
9/16/2012	Oysters	4	5.1
9/16/2012	Oysters	4	5.7
9/16/2012	Oysters	4	6.0

9/16/2012	Oysters	4	6.3
9/16/2012	Oysters	4	5.8
9/16/2012	Oysters	4	7.5
9/16/2012	Oysters	4	5.6
9/16/2012	Oysters	4	7.6
9/16/2012	Oysters	4	8.5
9/16/2012	Oysters	4	9.5
9/16/2012	Oysters	4	10.3
9/16/2012	Oysters	4	10.6
9/16/2012	Oysters	4	10.0
9/16/2012	Oysters	4	9.8
9/24/2012	Mesh	1	5.4
9/24/2012	Mesh	1	5.7
9/24/2012	Mesh	1	5.8
9/24/2012	Mesh	1	7.8
9/24/2012	Mesh	2	2.7
9/24/2012	Mesh	2	5.6
9/24/2012	Mesh	2	5.3
9/24/2012	Mesh	2	7.2
9/24/2012	Mesh	2	5.8
9/24/2012	Mesh	2	6.3
9/24/2012	Mesh	2	8.4
9/25/2012	Mesh	3	6.1
9/25/2012	Mesh	3	3.1
9/25/2012	Mesh	4	4.5
9/25/2012	Mesh	4	7.1
9/25/2012	Mesh	4	5.2
9/25/2012	Mesh	4	6.5
9/25/2012	Mesh	4	9.0
9/25/2012	Mesh	4	12.3
9/24/2012	Shells	1	5.8
9/24/2012	Shells	1	3.5
9/24/2012	Shells	1	5.7
9/24/2012	Shells	1	5.0
9/24/2012	Shells	1	7.2
9/24/2012	Shells	1	5.2
9/24/2012	Shells	1	5.6
9/24/2012	Shells	1	12.0
9/24/2012	Shells	2	2.2
9/24/2012	Shells	2	5.7
9/24/2012	Shells	2	6.0
9/24/2012	Shells	2	5.5
9/24/2012	Shells	2	5.2
9/24/2012	Shells	2	5.9
9/24/2012	Shells	2	6.3
9/24/2012	Shells	2	4.4
9/24/2012	Shells	2	5.6

9/24/2012	Shells	2	8.2
9/24/2012	Shells	2	10.0
9/24/2012	Shells	2	11.9
9/24/2012	Shells	2	1.4
9/25/2012	Shells	3	3.9
9/25/2012	Shells	3	5.6
9/25/2012	Shells	3	6.0
9/25/2012	Shells	3	7.1
9/25/2012	Shells	3	5.9
9/25/2012	Shells	3	8.9
9/25/2012	Shells	3	9.5
9/25/2012	Shells	3	14.7
9/25/2012	Shells	4	3.6
9/25/2012	Shells	4	5.6
9/25/2012	Shells	4	5.8
9/25/2012	Shells	4	6.1
9/24/2012	Oysters	1	4.1
9/24/2012	Oysters	1	5.2
9/24/2012	Oysters	1	5.4
9/24/2012	Oysters	1	5.9
9/24/2012	Oysters	1	9.9
9/24/2012	Oysters	1	6.2
9/24/2012	Oysters	1	6.3
9/24/2012	Oysters	1	6.5
9/24/2012	Oysters	1	9.7
9/24/2012	Oysters	2	2.8
9/24/2012	Oysters	2	2.5
9/24/2012	Oysters	2	2.5
9/24/2012	Oysters	2	4.1
9/24/2012	Oysters	2	4.5
9/24/2012	Oysters	2	5.9
9/24/2012	Oysters	2	5.6
9/24/2012	Oysters	2	5.7
9/24/2012	Oysters	2	5.1
9/24/2012	Oysters	2	5.4
9/24/2012	Oysters	2	6.6
9/24/2012	Oysters	2	8.0
9/24/2012	Oysters	2	8.6
9/24/2012	Oysters	2	8.0
9/24/2012	Oysters	2	9.4
9/24/2012	Oysters	2	10.0
9/24/2012	Oysters	2	5.1
9/24/2012	Oysters	2	10.2
9/24/2012	Oysters	2	10.3
9/24/2012	Oysters	2	11.6
9/24/2012	Oysters	2	12.2
9/24/2012	Oysters	2	12.3

9/25/2012	Oysters	3	6.3
9/25/2012	Oysters	3	7.5
9/25/2012	Oysters	3	8.0
9/25/2012	Oysters	3	4.6
9/25/2012	Oysters	3	4.8
9/25/2012	Oysters	3	5.2
9/25/2012	Oysters	3	4.9
9/25/2012	Oysters	3	6.3
9/25/2012	Oysters	3	5.6
9/25/2012	Oysters	3	6.1
9/25/2012	Oysters	3	7.0
9/25/2012	Oysters	3	7.5
9/25/2012	Oysters	3	8.9
9/25/2012	Oysters	3	7.2
9/25/2012	Oysters	3	10.3
9/25/2012	Oysters	3	11.6
9/25/2012	Oysters	4	6.2
9/25/2012	Oysters	4	13.4
9/25/2012	Oysters	4	4.7
9/25/2012	Oysters	4	4.5
9/25/2012	Oysters	4	5.4
9/25/2012	Oysters	4	5.5
9/25/2012	Oysters	4	5.9
9/25/2012	Oysters	4	5.6
9/25/2012	Oysters	4	5.9
9/25/2012	Oysters	4	5.2
9/25/2012	Oysters	4	6.3
9/25/2012	Oysters	4	8.1
9/25/2012	Oysters	4	10.6
9/25/2012	Oysters	4	9.9
9/25/2012	Oysters	4	10.5
9/25/2012	Oysters	4	12.2
9/25/2012	Oysters	4	10.3
